# Phosphorylation of histone H3 on Ser10 by auto-phosphorylated PAK1 is not essential for chromatin condensation and meiotic progression in porcine oocytes

**DOI:** 10.1186/2049-1891-4-13

**Published:** 2013-03-22

**Authors:** Bingyuan Wang, Wei Ma, Xiaoling Xu, Chao Wang, Yubo Zhu, Na An, Lei An, Zhonghong Wu, Jianhui Tian

**Affiliations:** 1 Ministry of Agriculture Key Laboratory of Animal Genetics, Breeding and Reproduction; National Engineering Laboratory for Animal Breeding, College of Animal Science and Technology, China Agricultural University, Beijing, 100193, P. R. China; 2 Institute of Animal Husbandry and Veterinary Medicine, Beijing Municipal Academy of Agriculture and Forestry Sciences, Beijing, 100097, P. R. China; 3 Department of Histology and Embryology; School of Basic Medical Sciences, Capital Medical University, Beijing, 100069, P. R. China

**Keywords:** Chromosome condensation, H3^Ser10^, Meiotic progression, PAK1^Thr423^, Porcine oocyte

## Abstract

**Background:**

The p21-activated kinase 1 (PAK1) is essential for mitosis and plays an important role in the regulation of microtubule assembly during oocyte meiotic maturation in mice; however, little is known about its role in porcine oocytes.

**Result:**

Total p21-activated kinase 1 (PAK1) and phosphorylated PAK1 at Thr423 (PAK1^Thr423^) were consistently expressed in porcine oocytes from the germinal vesicle (GV) to the second metaphase (MII) stages, but phosphorylation of histone H3 at Ser10 (H3^Ser10^) was only expressed after the GV stage. Immunofluorescence analysis revealed that PAK1^Thr423^ and H3^Ser10^ colocalized on chromosomes after the GV stage. Blocking of endogenous PAK1^Thr423^ by injecting a specific antibody decreased the phosphorylation level of H3^Ser10^; however, it had no impact on chromatin condensation, meiotic progression, cleavage rate of blastomeres or the rate of blastocyst formation.

**Conclusion:**

Phosphorylation of PAK1^Thr423^ is a spontaneous activation process and the activated PAK1^Thr423^ can promote the phosphorylation of H3^Ser10^; however, this pathway is not required for meiotic maturation of porcine oocytes or early embryonic development.

## Background

At birth, mammalian oocytes are arrested at prophase I when the nucleus is referred to as a germinal vesicle (GV), in which the chromatin is not condensed. During oocyte growth, chromatin in the GV condenses into perinucleolar rings [1]. After being stimulated by a preovulatory gonadotropin surge or when released from their surrounding follicular cells into suitable culture conditions, oocytes spontaneously resume meiosis, GV breakdown (GVBD) occurs and the chromatin is condensed into chromosomes.

Chromosome condensation, as the first visible process during oocyte maturation, is essential for the correct packaging of chromatin fibers into chromosomes and their proper segregation during meiotic maturation. Recent studies have shown that histone modifications during oocyte development are crucial for oocyte maturation in mammals and that disruption of these modifications leads to defective chromosome condensation and segregation, inevitably leading to delayed maturation [2]. Histone H3 is one of the core histones bound to DNA in the nucleosomes and the phosphorylation of histone H3 at serine 10 (H3^Ser10^) has been characterized extensively [3-8]. In SKN and HeLa cells, H3^Ser10^ regulates protein–protein interactions that drive and coordinate chromatin condensation as cells enter the M-phase of mitosis [3]. During meiosis in the mouse ovary, the dynamic expression of H3^Ser10^ has been related to changes in chromatin condensation [6]. In pig oocytes, a low level of H3^Ser10^ is observed in GVs, which dramatically increased in all chromosomes from pro-metaphase I (Pro-MI) to the second metaphase (MII) [7]. Despite the dynamic expression of H3^Ser10^ and its localization on chromatin, H3^Ser10^ was not found to be essential for chromatin condensation in pig oocytes; however, it might be required for further processing of chromosomes during meiosis [9].

It could be speculated that H3^Ser10^ plays a different role during oocyte meiosis between mice and pigs, but evidence is needed to determine the definite function of H3^Ser10^ in pig oocytes. Phosphorylation of H3^Ser10^ can be regulated by multiple kinases [10,11]; for example, Aurora B has been shown to be an important kinase *in vivo*[12]. Inhibition of Aurora B significantly decreases the level of H3^Ser10^ in mouse oocytes, resulting in chromosome misalignment [13]. In maturing porcine oocytes, activation of Aurora B precedes the phosphorylation of H3^Ser10^[9]. Moreover, treatment of immature porcine oocytes with the protein phosphatase 1/2a (PP1/2a) inhibitors, okadaic acid and calyculin A, induced rapid chromosome condensation with hyperphosphorylation of H3^Ser10^[14]. Whether H3^Ser10^ is directly responsible for catalyzing chromatin condensation during porcine oocyte meiosis, or if any other kinases are involved in this process, remains to be elucidated as the underlying mechanisms are not fully understood.

The p21-activated kinase (PAK) family belongs to a group of serine/threonine kinases, which have been identified as targets of the Rho GTPases Rac1 and Cdc42 [15,16]. The PAK family includes six PAK isoforms (PAK 1–6), which are composed of an N-terminal p21 GTPase-binding domain and a C-terminal kinase domain [17]. Specifically, PAK1 contains a PAK auto-inhibitory domain in the N-terminal regulatory domain, which can inhibit kinase activation by interaction with the catalytic domain [18,19]. Phosphorylation of PAK1 on threonine 423 (PAK1^Thr423^), is a key event in PAK1 activation and is important for maintaining its relief from auto-inhibition [20]. The activated form of PAK1 behaves like a chromosomal passenger protein and can interact with and phosphorylate H3^Ser10^[21].The literature suggests that the PAK1-histone H3 pathway is potentially involved in regulating mitotic events, such as chromatin condensation and subsequent chromosomal capture, movement and segregation [21]. It is not fully understood whether PAK1-mediated phosphorylation of histone H3 is conserved in mammalian oocytes during meiosis. Indeed, recent studies have shown that depleted expression of PAK1 in mouse oocytes lead to defects in meiotic spindle assembly, chromosome alignment and polar body extrusion, but the functional mechanism was not presented and needs to be clarified [22].

Given the uncertainty on the importance of the PAK1-histone H3 pathway in oocyte maturation, we examined the expression and subcellular distribution of PAK1^Thr423^ and its relationship with H3^Ser10^ in porcine oocytes during meiotic maturation. Our results provide strong evidence that the phosphorylation of histone H3^Ser10^ is regulated by PAK1^Thr423^; however, this regulation is not required for oocyte chromatin condensation and meiotic progression, or for subsequent embryonic development.

## Materials

### Chemicals and antibodies

All chemicals were purchased from Sigma-Aldrich (St. Louis, MO, USA) unless otherwise indicated. The rabbit polyclonal anti-PAK1 antibody was purchased from Signalway Antibody Co. (Ab-212; Pearland, TX, USA), the rabbit polyclonal anti-phosphorylated PAK1 (Thr423) antibody was purchased from Abgent Primary Antibody Co. (San Diego, CA, USA) and the monoclonal mouse anti-phosphorylated Histone H3 (Ser10) antibody was purchased from Millipore Corp. (Billerica, MA, USA).

### Porcine oocyte collection and culture

Ovaries of prepubertal gilts were collected at a local commercial slaughterhouse. Porcine cumulus cell–oocyte complexes were aspirated from the antral follicles in the ovaries as described [23]. Cumulus cell–oocyte complexes with evenly granulated cytoplasm were selected and cultured in TCM-199 medium (Gibco, Grand Island, NY, USA) containing 10% porcine follicular fluid, 0.1 mg/mL l*-*cysteine, 10 ng/mL epidermal growth factor, 10 IU/mL equine chorionic gonadotropin and 10 IU/mL human chorionic gonadotropin at 38.5°C in 100% humidity under an atmosphere of 5% CO_2_ in air.

### Western blotting

One hundred porcine oocytes released from cumulus cells were collected at different time points and frozen in 2 × Laemmli sample buffer (Bio-Rad, Hercules, CA, USA) containing protease inhibitors. Prior to analysis, the samples were thawed and subsequently heated to 100°C for 5 min. The proteins were then separated on a 12% polyacrylamide gel containing 0.1% sodium dodecyl sulfate and then transferred onto a poly(vinylidene fluoride) membrane (Amersham, Piscataway, NJ, USA). The membranes were blocked in Tris-buffered saline containing 0.05% (v/v) Tween-20 with 5% non-fat dried milk overnight at 4°C and incubated with polyclonal rabbit anti-PAK1 antibody (1:1,000), polyclonal rabbit anti-phosphorylated PAK1 (Thr423) antibody (1:1,000) or monoclonal mouse anti-phosphorylated Histone H3 (Ser10) antibody (1:500) for 2 h at room temperature. Finally, peroxidase-conjugated secondary antibody (Jackson ImmunoResearch, West Grove, PA, USA) was added for 1 h and protein bands were then detected using an ECL-plus system (Amersham).

### Immunofluorescence analysis and confocal microscopy

Oocytes at different time points were fixed in 2% paraformaldehyde in phosphate buffered saline (PBS) for at least 30 min at room temperature and then incubated in PBS plus 1% Triton X-100 for 30 min at room temperature, followed by blocking in 1% BSA at 4°C overnight. The oocytes were incubated for 2 h at 37°C with monoclonal anti-phosphorylated histone H3 (Ser 10) antibody (1:500 dilution), polyclonal rabbit anti-phosphorylated PAK1 (Thr423) antibody (1:1,000 dilution) or anti-acetylated tubulin antibody (1:10,000 dilution). The oocytes were then labeled with Alexa Fluor 594-labeled Goat anti-Rabbit or Alexa Fluor 488-labeled Goat anti-Mouse (Molecular Probes, Eugene, OR, USA) secondary antibodies for 1 h at room temperature in the dark. After washing, the oocytes were stained with [4′], 6-diamidino-2-phenylindole **(**DAPI; Sigma-Aldrich) to detect DNA and examined using a confocal laser scanning microscope (Zeiss LSM 510 META, Carl Zeiss GmbH, Jena, Germany).

### Chromosomal spreading analysis

The zona pellucida of oocytes was removed by exposure to acid Tyrode’s solution (pH 2.5). Zona-free oocytes then were fixed by carefully placing them onto a microscope slide dipped in a solution of 1% paraformaldehyde in distilled H_2_O containing 0.5% Triton X-100 [6]. To analyze the localization of PAK1^Thr423^ and H3^Ser10^ on chromosomes, the slides were incubated with polyclonal rabbit anti-phosphorylated PAK1 (T423) antibody (1:500) and monoclonal mouse anti-phosphorylated Histone H3 (Ser10) antibody (1:250) in PBS for 1 h at 37°C. This was followed by incubation with Alexa Fluor 594-labeled goat anti-rabbit IgG (1:500 dilution) and Alexa Fluor-labeled 488 goat anti-mouse IgG secondary antibodies (Molecular Probes) for 1 h at room temperature. After the chromosomes had been counterstained with DAPI, the samples were analyzed by an investigator blinded to their treatment group, at 1000 × magnification using a Zeiss LSM 510 META microscope (Carl Zeiss GmbH, Jena, Germany).

### Antibody microinjection into oocytes

Anti-PAK1^Thr423^ antibody (stock solution, 100 μg/mL) was injected into the cytoplasm of zona-intact denuded oocytes at the GV stage, with normal rabbit IgG-injected oocytes and non-injected oocytes used as controls [24]. Sterile Femtotip capillaries and a FemtoJet microinjector (Eppendorf, Westbury, NY, USA) were used to standardize the injection volumes; an injection volume of ~5 pL per oocyte was used in all experiments. Ten micrograms of cycloheximide (CHX) per milliliter was added to the manipulation medium to prevent GVBD. After microinjection, oocytes were washed thoroughly with TCM-199 medium and cultured in fresh medium under an atmosphere of 5% CO_2_ in air at 38.5°C.

### Parthenogenetic activation of oocytes and embryo culture

After 42–44 h of culture, the cumulus cells were removed physically from the oocytes using a narrow pipette combined with 0.1% hyaluronidase. Oocytes with a first polar body were selected and placed between 0.2 mm diameter platinum electrodes 1 mm apart in fusion activation medium and then subjected to a single DC pulse for 30 μs (1,500 V/cm; ECM 2001, BTX Inc., San Diego, CA, USA) [25]. Activated oocytes were then immediately transferred into embryo culture medium PZM3 supplemented with 7.5 μg/mL cytochalasin B and 10 μg/mL CHX and cultured for 4 h. Embryos were then cultured in groups of 15–20 per 100 μL of PZM3 under sterile mineral oil for 3 days (cleavage stage) or 7 days (blastocyst stage) at 38.5°C under 5% CO_2_ in air.

### Statistical analysis

All data are presented as the mean ± SEM, determined from a minimum of three independent experimental replicates. Data were analyzed by one-way analysis of variance (ANOVA) using SAS 9.2 software (SAS Institute, Cary, NC, USA) and *P* < 0.05 was considered statistically significant.

## Results

### Expression and subcellular localization of PAK1^Thr423^ during porcine oocyte meiotic maturation

**Figure 1:**
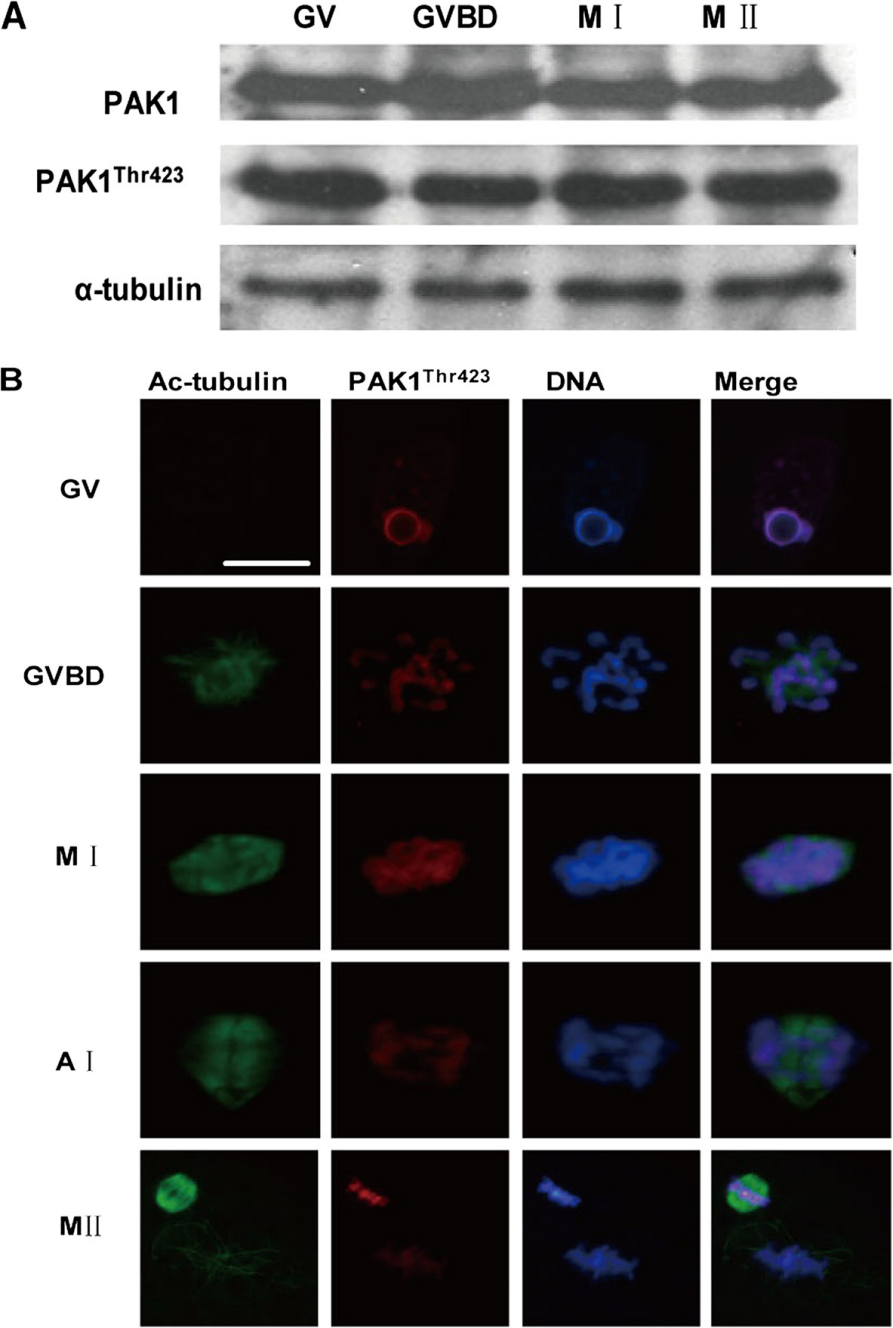
**The expression level and localization of PAK1**^**Thr423 **^**in porcine oocytes during meiotic maturation.** (**A**) A representative western blot showing the expression level of PAK1 and PAK1^Thr423^ at the GV, GVBD, MI and MII stages of oocyte maturation is shown. (**B**) Representative images of Ac-tubulin (green), PAK1^Thr423^ (red) and DAPI (blue) immunostaining in GV, GVBD, MI, AI and MII oocytes. Bar = 10 μm.

To investigate the role of PAK1 and PAK1^Thr423^ during porcine oocyte meiotic maturation, we examined their expression by western blotting. We analyzed porcine oocytes at 0, 18, 30 and 44 h, corresponding to the GV, GVBD, MI and MII stages, respectively. As shown in Figure 1A, high and stable levels of PAK1 and PAK1^Thr423^ expression were detected from the GV to the MII stages.

To examine the subcellular localization of PAK1^Thr423^, we performed immunofluorescent staining of porcine oocytes at different stages of maturation. As shown in Figure 1B, PAK1^Thr423^-immunopositive staining was initially detected around the nucleolus at the GV stage and then was closely colocalized with the chromosomes from the pro-MI to MII stage. Taken together, our immunofluorescence analysis and western blot results were consistent and revealed that the pattern of PAK1^Thr423^ distribution was closely related to meiotic progression, especially nuclear dynamics, in porcine oocytes.

### Expression of H3^Ser10^ and its colocalization with PAK1^Thr423^ in porcine oocytes during meiotic maturation

**Figure 2:**
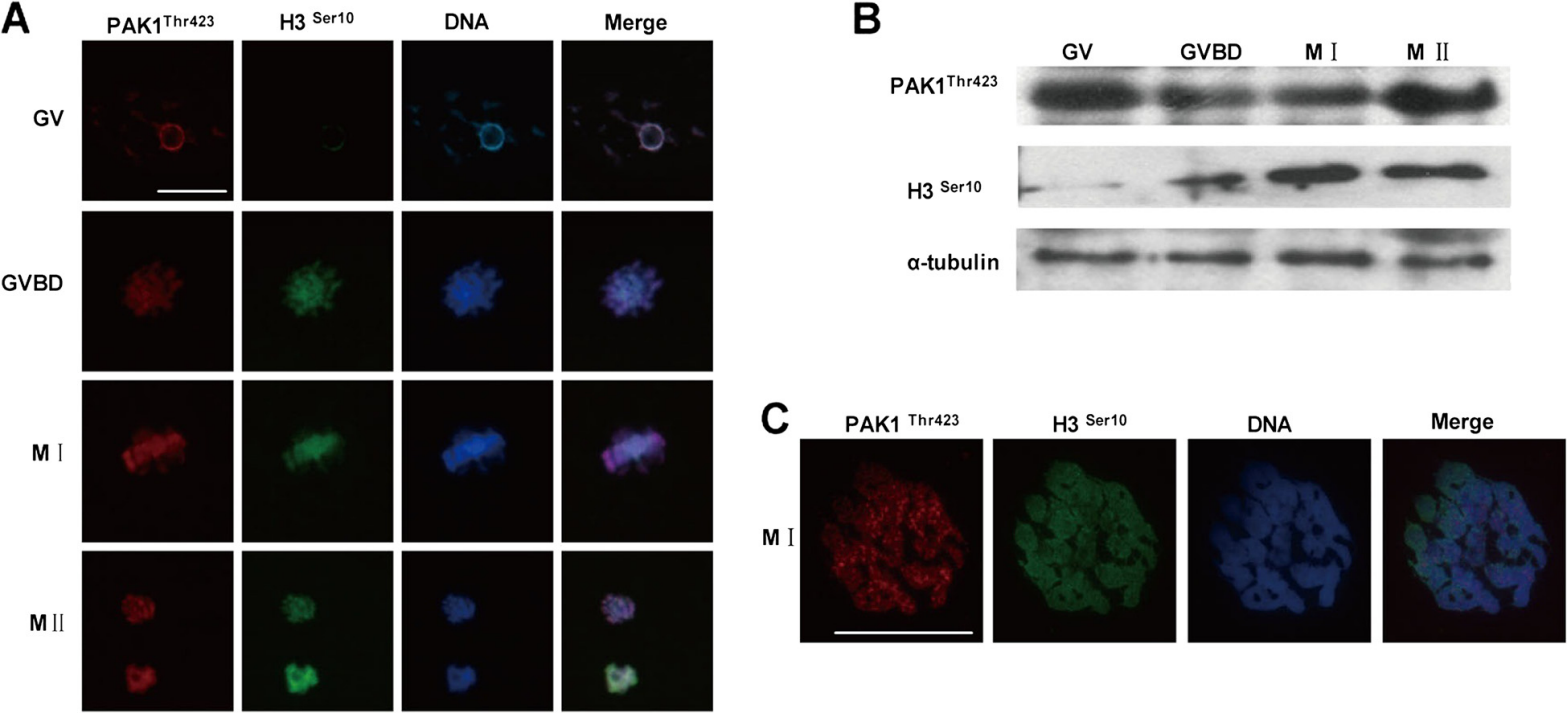
**The expression and subcellular localization of PAK1**^**Thr423 **^**and histone H3**^**Ser10 **^**during porcine oocyte meiotic maturation.** (**A**) Representative images of PAK1^Thr423^ (red), H3^Ser10^ (green) and DAPI (blue) immunostaining in GV, GVBD, MI and MII oocytes. (**B**) A representative western blot showing the expression level of PAK1^Thr423^ and H3^Ser10^ at the GV, GVBD, MI and MII stages of oocyte maturation. (**C**) Representative images of chromosomal spreads showing MI oocytes costained with H3^Ser10^ (green), PAK1^Thr423^ (red) and DAPI (blue). Bar = 10 μm.

It has been demonstrated that PAK1^Thr423^ phosphorylates the downstream factor H3^Ser10^ in human breast cancer cells [21]. Here, we examined the temporal and spatial distribution of H3^Ser10^, as well as its association with PAK1^Thr423^ at different stages of porcine oocyte meiosis. As shown in Figure 2A, immunofluorescent staining confirmed that the expression of PAK1^Thr423^ preceded that of H3^Ser10^ in oocytes. Compared with PAK1^Thr423^, only faint H3^Ser10^ expression was observed at the GV stage; however, by the GVBD stage H3^Ser10^ expression had increased dramatically and it colocalized with PAK1^Thr423^ and the chromosomes. Specifically, it appeared that H3^Ser10^ was concentrated on condensing chromatin and individual chromosomes during the pro-MI to MII stages. These results were consistent with results from our western blot analysis (Figure 2B), which revealed an extremely low level of H3^Ser10^ expression at the GV stage that then increased progressively at the MI and MII stages. As in the results of Figure 1A, we also demonstrated a high and stable expression level of PAK1^Thr423^ from the GV to MII stage. Through our analysis of chromosomal spreading, we verified that PAK1^Thr423^ colocalizes with H3^Ser10^ on chromosomes in porcine oocytes (Figure 2C). Taken together, these results indicate a unique temporal and spatial correlation in the expression patterns of PAK1^Thr423^ and H3^Ser10^.

### Blocking PAK1^Thr423^ leads to reduced expression of H3^Ser10^, but has no impact on chromosome configuration in porcine oocytes

**Figure 3:**
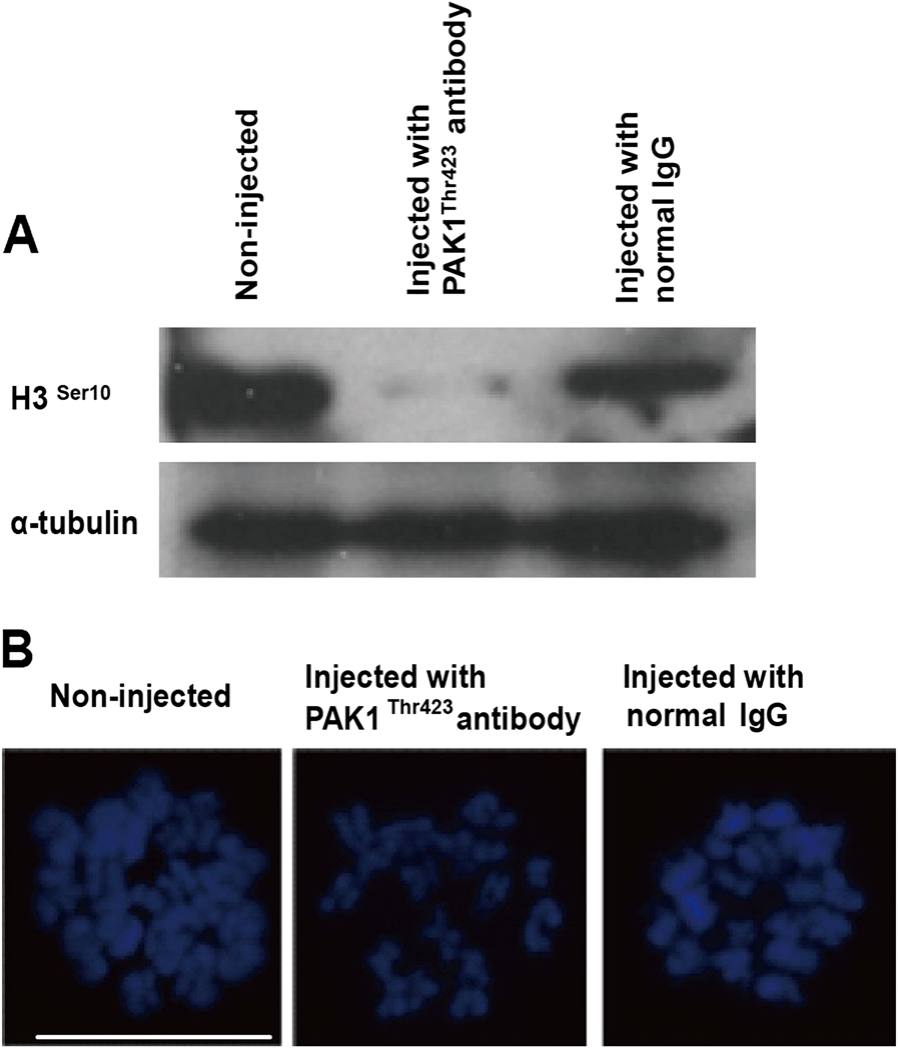
**Effects of PAK1**^**Thr423 **^**inactivation on the expression of H3**^**Ser10 **^**and chromosome configuration in porcine oocytes.** (**A**) Representative western blot of protein extracted from porcine oocytes microinjected with an anti-PAK1^Thr423^ antibody and controls, showing the resultant decline in H3^Ser10^ expression. (**B**) Representative images of DAPI staining (blue) following chromosomal spreading in control and anti-PAK1^Thr423^ antibody injected embryos. Bar = 10 μm.

To block PAK1^Thr423^ activity, we microinjected an anti-PAK1^Thr423^ antibody into the cytoplasm of GV stage porcine oocytes, then analyzed the effects on H3^Ser10^ expression and oocyte maturation. Our results showed that the expression level of H3^Ser10^ was dramatically reduced in the antibody-injected oocytes compared with the control oocytes (Figure 3A), implying that PAK1^Thr423^ activity is required for the phosphorylation of H3^Ser10^.

To determine whether the reduction in H3^Ser10^ expression following anti-PAK1^Thr423^ antibody injection had an effect on chromosome configuration, the chromosome structure was assayed after 44 h in culture (Figure 3B). As shown in Table 1, there was no statistically significant effect of anti-PAK1^Thr423^ antibody injection on chromosome structure and formation, although there were fewer oocytes with a normal chromosome configuration in the anti-PAK1^Thr423^ antibody-injected group0.777 ± 0.041, compared with the two control groups (rabbit IgG-injected and non-injected groups; 0.819 ± 0.028 and 0.833 ± 0.051, respectively).

**Table 1:** **Effects of PAK1**^**Thr423 **^**inactivation on chromosome formation, meiotic progression and embryonic development in porcine oocytes**

**Treatment**	**No. of normal oocytes (%)**	**No. (%)of oocytes at stage of**		**No. of cleaved embryos (%)**	N**o. of blastocysts(%)**
		**MI**	**MII**		
Non-injected	76 (83.3 ± 5.1)^a^	79 (24.7 ± 12.3) ^a^	207 (65.8 ± 10.1) ^a^	229 (91.3 ± 8.0) ^a^	80(28.1 ± 6.6) ^b^
Injected with normal IgG	81 (81.9 ± 2.8)^a^	82 (24.6 ± 9.7) ^a^	216 (65.5 ± 10.0) ^a^	221 (90.3 ± 4.6) ^a^	62 (25.0 ± 7.2) ^b^
Injected with PAK1^Thr423^ antibody	97 (77.7 ± 4.1)^a^	124 (30.4 ± 7.2) ^a^	251 (59.1 ± 8.0) ^a^	266 (88.6 ± 4.0) ^a^	54 (18.6 ± 8.1) ^b^

The values are the mean ± SEM of five independent replicates. Within the same column, values with different superscript letters are significantly different (*P* < 0.05).

### Loss of PAK1^Thr423^ activity has no influence on oocyte meiotic progression and early embryo development

We next investigated whether the reduction in H3^Ser10^ expression following antibody injection had any effect on oocyte meiotic progression and early embryo development. As shown in Table 1, although there was a trend for a higher percentage of oocytes at the MI stage and a lower percentage of oocytes at the MII stage following anti-PAK1^Thr423^ antibody injection, compared with the control groups, these differences were not statistically significant. Similarly, activated oocytes were cultured for 3 and 7 days in PZM3 medium and the rates of embryo cleavage and blastocyst formation were assessed (Table 1). Oocytes injected with the anti-PAK1^Thr423^ antibody had lower rates of embryo cleavage and blastocyst formation compared with control oocytes; however, these differences were not statistically significant. Taken together, this data implies that PAK1^Thr423^ activity might be not essential for meiotic progression in porcine oocytes.

## Discussion

In this study, we provide evidence for the first time that the activity of autophosphorylated PAK1^Thr423^ contributes to the phosphorylation of Histone H3^Ser10^ during porcine oocyte meiotic maturation. Although PAK1^Thr423^ and H3^Ser10^ were perfectly colocalized on the chromosomes, the blockade of PAK1^Thr423^ and consequent reduction in H3^Ser10^ expression had no significant effect on chromosome configuration, meiotic progression or early embryo development.

Chromatin condensation is the first step in oocyte nuclear maturation and is required for the correct segregation of chromosomes during meiosis. The phosphorylation of histone H3 is thought to be involved in chromatin condensation, chromosome congregation and segregation during mitotic cell division and oocyte meiosis [26-28]. The phosphorylation of H3^Ser10^ has been suggested to play a key role in this process [4,5]. For example, Wang et al. observed that H3^Ser10^ colocalized with chromatin in the GV at prophase I of meiosis in mouse oocytes, and then became distributed along entire chromosomes with intensive aggregation in the pericentromeric heterochromatin from the pro-MI to MII stages [13]. However, Swain et al. could not detect any H3^Ser10^ expression in mouse oocytes at the GV stage [7]. During porcine oocyte maturation, a low level of H3^Ser10^ has been observed at the GV stage, which significantly increased upon GVBD; furthermore, H3^Ser10^ was also shown to tag intact chromosomes from the pro-MI to MII stages [2,7,9]. These results are in agreement with our findings, as only extremely low levels of H3^Ser10^ expression were detected at the GV stage, but it increased dramatically at the MI and MII stages. The concurrence of elevated H3^Ser10^ and chromatin remodeling upon GVBD implies that the activity of H3^Ser10^ might be required for chromatin condensation into individual chromosomes.

Histone H3 can be phosphorylated by several kinases. Thus, Aurora B is required for histone H3 phosphorylation during mitosis in *Drosophila*[29] and *Xenopus*[30] and plays a central role in the assembly and maintenance of mitotic chromosome structure. As a substrate of PAK1^Thr423^, histone H3 can be phosphorylated at Ser10 in human breast cancer cells [21]. In the current study, immunofluorescence analysis and western blotting confirmed that autophosphorylation of PAK1^Thr423^ preceded phosphorylated H3^Ser10^ expression during porcine oocyte maturation, and that PAK1^Thr423^ was stably expressed and colocalized with H3^Ser10^ on chromosomes. We also found that the expression of H3^Ser10^ was decreased in porcine oocytes microinjected with an antibody against PAK1^Thr423^, indicating that the activity of PAK1^Thr423^ regulates the H3^Ser10^, which is consistent with findings in human cancer cells [21].

Given these prior findings, we hypothesized that PAK1^Thr423^ might play a role in chromatin remodeling and separation in porcine oocyte meiosis by phosphorylating H3^Ser10^. Unfortunately, this hypothesis had to be rejected based on our results, as oocytes microinjected with anti-PAK1^Thr423^ antibodies displayed no significant changes in chromosome configuration, rates of meiotic progression or subsequent early embryonic development, despite having a reduced level of H3^Ser10^ expression.

Bui et al. [14] claimed that the activity of H3^Ser10^ was related to chromatin remodeling in porcine oocytes. Similarly, increased H3^Ser10^ expression following treatment with a PP1/2a inhibitor was associated with chromatin condensation in mouse oocytes [6]. Despite these data, our results indicated that H3^Ser10^ was not essential in these processes. In agreement with this conclusion, Jelínková et al. found that H3^Ser10^ was not essential for chromatin condensation to chromosomes upon the resumption of meiosis in porcine oocytes [9]. The studies of Bui et al. and Swain et al. both used chemical inhibitors to alter the intracellular level of H3^Ser10^ in oocytes. Thus, it is hard to rule out the possibility that other kinases or pathways essential for chromatin remodeling were affected [6,14]. Accordingly, such nonspecific effects could have affected chromatin structure independently of the effect on H3^Ser10^. In our study, the depletion of H3^Ser10^ by microinjection with a specific antibody against PAK1^Thr423^ provided a more targeted approach. Thus, in agreement with Jelínková et al., we believe the activity of H3^Ser10^ does not play an essential role in chromatin remodeling during meiosis in pig oocytes [9].

## Conclusions

Our results clearly demonstrate that PAK1^Thr423^ regulates the phosphorylation of H3^Ser10^ in porcine oocytes. Despite this interaction, our results suggest that the PAK1^Thr423^-H3^Ser10^ pathway is not essential for the maintenance of chromosome configuration and meiotic progression in pig oocytes, or for early embryo development.

## Competing interests

The authors declare that they have no competing interests.

## Authors’ contributions

BYW as the lead author designed the research protocol, carried out the experiments, collected and analyzed data and drafted the manuscript. WM was instrumental in guiding the experiment skills. XLX, CW, YBZ and NA participated in carrying out all the lab work, data collection and analysis. WM, LA, ZHW and JHT gave significant contributions to designing the study. All authors have read and approved the final manuscript.
